# THIS IS THE YEAR THAT WAS: THE BEST OF THE JOURNAL OF GERONTOLOGY BIOLOGICAL SCIENCES

**DOI:** 10.1093/geroni/igad104.0062

**Published:** 2023-12-21

**Authors:** Gustavo Duque

## Abstract

The Journal of Gerontology Biological Sciences (JGBS) is one of the Gerontological Society of America publications. It publishes articles on the biological aspects of aging in areas such as translational gerontology, biomarkers of aging, biochemistry, biodemography, cellular and molecular biology, and geroscience. Biological insights are also published in studies from invertebrates, vertebrates, and mammalian species, including humans. With an impact factor of 6.59, this journal is one of the leading journals in the field. Overall, and although the journal is highly valued by biomedical researchers worldwide, the authors of our top and most impactful papers do not usually have the opportunity to answer the questions from their peers constructively and interactively. In addition, there is a significant number of biomedical researchers that, although members of the Society, do not submit their best results to the journal. This symposium, chaired by the Editor-in-Chief of the JGBS, will comprise six presentations by the authors of the most influential papers of the year (June 2022 to June 2023). The papers will be selected based on their impact via citations and/or social media. Presenters will be chosen following diversity, equity, and inclusion principles. Various topics going from cells to animal models will be covered while highlighting the translational potential of those discoveries. Ten minutes presentation will be followed by 5 minutes of Q&A with ample opportunity to interact.

